# Distal Metatarsal Articular Angle and First Metatarsal Pronation: A Weightbearing CT Correlation Study

**DOI:** 10.1177/10711007251412444

**Published:** 2026-02-19

**Authors:** Arvind Vijapur, Mohammed Shaath, Shelain Patel, Nicholas Cullen, Karan Malhotra, Matthew Welck

**Affiliations:** 1 Foot & Ankle unit, Royal National Orthopaedic Hospital, Stanmore, Middlesex, UK; 2 Department of Ortho & MSK Science, University College London, London, UK

**Keywords:** hallux valgus, metatarsal pronation, DMAA, weight bearing CT

## Abstract

**Background:**

The distal metatarsal articular angle (DMAA) is a measurement used in surgical decision making of hallux valgus correction. However, it is difficult to measure on plain radiographs, is subject to projection bias, and its role in pathology is unclear. With the advent of weight-bearing CT (WBCT), our understanding of hallux valgus as a multiplanar deformity has evolved. The aim of this study was to investigate whether there is a relationship between the DMAA and pronation of the first metatarsal head in patients with hallux valgus.

**Methods:**

This was a single-centre, retrospective analysis of 50 patients with hallux valgus deformity who had WBCTs obtained as part of routine pre-operative work-up. Patients with metatarsophalangeal joint arthritis, hindfoot deformity, and previous surgery were excluded. From the WBCT images, digital radiographs were created and the DMAA measured. Measurements were taken by 2 authors, each repeated twice and the average of all 4 measurements used in analysis. We also measured intermetatarsal angle (IMA), hallux valgus angle (HVA), and metatarsal pronation angle (MPA).

**Results:**

There were 41 females and 9 males, with a mean age of 52.4 ± 15.8 years. The IMA was 14.5 ± 3.3 degrees, HVA was 29.3 ± 8.4 degrees, MPA was 11.7 ± 6.3 degrees, and DMAA was 15.5 ± 5.3 degrees. Intraclass correlation coefficient (ICC) for intra-observer reliability was 0.829 for assessor 1 and 0.910 for assessor 2. ICC for inter-observer reliability was 0.727. Pearson correlation revealed no link between IMA and DMAA, nor HVA and DMAA. However, there was a significant (albeit small) correlation between MPA and DMAA (*r* = 0.337, *P* = .017). In the mixed effects model, MPA remained an independent predictor of DMAA (β = 0.276, 95% CI 0.153-0.399, *P* < .001).

**Conclusion:**

There was reasonable reliability in measuring DMAA between authors on WBCT although there was variation in measurements. We found the DMAA appeared to increase with increasing metatarsal pronation. The DMAA may therefore be (in part) projection artefact secondary to metatarsal pronation, and surgeons should be aware of this during surgical planning.

**Level of Evidence:** Level IV, retrospective case series.

## Introduction

Hallux valgus (HV) is a complex, multiplanar deformity affecting the first ray.^1^ Traditionally the deformity is measured using weightbearing plain radiographs, with commonly used radiographic measurements including hallux valgus angle (HVA), intermetatarsal angle (IMA), and the distal metatarsal articular angle (DMAA). The DMAA is a 2-dimensional measurement obtained from standard dorsoplantar radiographs. It has long been thought of as a marker of the valgus orientation of the distal articular surface of the first metatarsal head that has influenced the selection of surgical techniques in hallux valgus correction. Where the DMAA requires addressing, it has classically been corrected with distal osteotomies that incorporate a medial closing wedge to rotate the metatarsal head in the axial plane.^2^ The normal values for the DMAA has been reported to vary from −3 to 26 degrees.^3^ However, the validity of this angle remains controversial, and studies have shown poor interobserver reliability, particularly in the presence of first metatarsal pronation.^3-7^ Furthermore, its value can be confused with the rounded lateral edge of the first metatarsal head, as described by Okuda et al,^8^ and which may indicate pronation.

Pronation of the first metatarsal refers to rotation where its plantar surface turns towards the second metatarsal. The precise cause of this pronation remains unclear. However, it is believed that the entire medial column contributes to this rotational change seen in hallux valgus.^9^ Accurate detection of this pronation remains challenging. Yamaguchi et al^10^ described a rounder shape of the first metatarsal head that correlated with metatarsal pronation. Although early attempts at assessing pronation used radiographs, they have several limitations as they are affected by projection artefacts and cannot assess coronal rotation of the first metatarsal. Therefore, radiographs may not fully identify the complexity of the deformity.^11,12^

Cross-sectional imaging is required for coronal plane assessment. The advent of weightbearing cone-beam CT (WBCT) has allowed us to assess anatomy in all 3 planes under physiological weightbearing. This has been crucial for advancing our understanding of lower limb deformities.^13^ WBCT has enabled us to accurately quantify the normal values for metatarsal pronation (metatarsal pronation angle) and examine its association with hallux valgus and other deformities in the foot and ankle.^14-18^ Although previous studies have used weightbearing CT to assess the DMAA, they have not examined the relationship to metatarsal pronation.^19^

This study aimed to evaluate the correlation of the DMAA and metatarsal pronation angle (MPA) in hallux valgus deformity using WBCT. The objective was to establish whether DMAA is in fact a 2-dimensional artefact of coronal plane pronation, rather than a true articular deformity. Our null hypothesis was that there is no correlation between DMAA and MPA.

## Methods

This was a single-centre, retrospective observational study. WBCT scans are done as part of our standard assessment for patients, including (more recently) patients with hallux valgus deformity. From 2019 onward, all patients presenting with hallux valgus had a WBCT as their main modality of imaging, instead of radiographs. A prospective database of all scans was maintained, and this was used to identify patients for this study. WBCT scans performed until 2020 were reviewed using the database. As this study only involved retrospective analysis of imaging data captured as part of routine care, no ethical approval was required. We applied strict inclusion and exclusion criteria to minimize confounding factors and selection bias. The inclusion criteria were patients aged 16 years and older with hallux valgus who had WBCT as part of routine preoperative evaluation. Exclusion criteria were previous foot surgery, metatarsophalangeal joint arthritis, or hindfoot deformity, as these could distort anatomical landmarks. No participants were excluded after meeting initial inclusion criteria. Only one foot was included per patient.

### Radiographic Measurements

Images were obtained using a PedCAT unit (CurveBeam LLC, Warrington, PA) in the outpatient department. Patients who had a WBCT scan did not also have a plain radiograph. Coronal, sagittal, and axial slices of the scans were used for measurements performed with a digital patient archive communication system (PACS). All data sets were anonymized. Measurements were performed independently by 2 foot and ankle–trained orthopaedic surgeons twice each to establish intraobserver and interobserver reliability. The assessors were masked to each other’s results. Measurements included the DMAA, the 1-2 IMA, the HVA, and the MPA.

**Figure 1.**
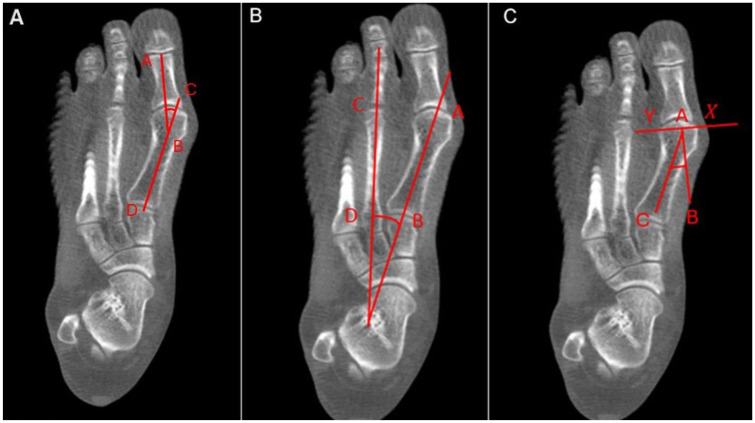
Panel A shows the measurement of the hallux valgus angle (HVA), and panel B shows the measurement of the 1-2 intermetatarsal angle (IMA). Panel C shows the measurement of the distal metatarsal articular angle (DMAA) on a digitally reconstructed radiograph.

The WBCT was oriented in the sagittal plane along the axis of the first metatarsal. Dorsoplantar digitally reconstructed radiographs were then created using a slab width of 50 mm. The HVA, distal metatarsal articular angle and IMA were measured on this axial plane (Figure 1).

### Measurement of DMAA

The DMAA purports to quantify the orientation of the distal articular surface of the first metatarsal relative to its longitudinal axis. To determine this angle, 2 points were identified at the most medial and lateral extents of the distal metatarsal articular surface (X and Y). A line connecting these points (X-Y) represents the lateral slope of the articular surface. A perpendicular line (A-B) is then constructed from this slope. The DMAA is defined as the angle formed between this perpendicular line (A-B) and the longitudinal diaphyseal axis of the first metatarsal (line A-C) as per previously published literature.^20^

### Measurement of Metatarsal Pronation Angle

**Figure 2.**
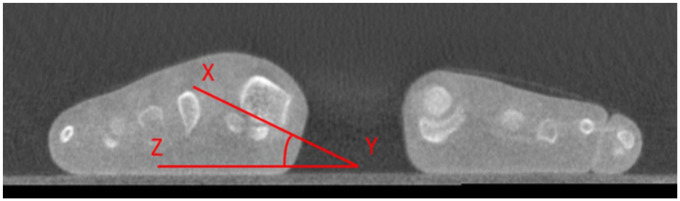
Metatarsal pronation angle (MPA) measurement technique.

To standardize axial imaging, a line perpendicular to the longitudinal axis of the third metatarsal was drawn to bisect the sesamoid complex. The corresponding 1-mm coronal slice at this level was then selected for measuring the MPA. The MPA was defined as the angle between 2 lines: one connecting the most inferomedial border of the medial sesamoid facet to the most inferolateral border of the lateral sesamoid facet (X-Y), and the other drawn along the weightbearing surface (Y-Z). This angle quantified the degree of metatarsal pronation. In accordance with the technique outlined by Kim et al^21^ and Najefi et al,^14-16^ pronation was recorded as a positive value (Figure 2).

### Statistical Analysis

Descriptive statistics (mean ± SD, range) were calculated for continuous variables. Data were normally distributed. Pearson correlation coefficient was used to assess relationships between DMAA and other angles (IMA, HVA, MPA). A *P* value of <.05 was considered statistically significant.

Reliability was evaluated using intraclass correlation coefficient (ICC). Reliability refers to the degree to which measurements can be consistently repeated. It consists of both correlation and agreement between repeated assessments.^22^ The higher reliability reflects less measurement error and more accurate results. The ICC is a preferred statistical measure because it represents both consistency and reproducibility simultaneously. ICC thresholds used were poor (less than 0.5), moderate (0.5-0.75), good (0.75-0.9), and excellent (greater than 0.9) reliability.^22^

The data was further analyzed with a mixed effects linear regression model constructed using DMAA as the dependent variable. The MPA was included as the main predictor, and IMA and HVA were included as covariates (fixed effects). The raters were included as a random effect to allow the model to account for variability between raters. Statistical analyses were performed using SPSS software (v29, IBM, Armonk, USA) and Python 3.12.3.

As this was a retrospective analysis of data that included all eligible cases, a power analysis was not performed.

## Results

A total of 50 patients who met the inclusion criteria were analyzed, of which 41 were females and 9 males. The mean age was 52.42 (range, 16-83) years. Mean values for measured variables are listed in Table 1.

**Table 1.** Summary of Values for Measured Angles.

Angle Measured	Minimum	Maximum	Mean	SD
Intermetatarsal angle	11.0	22.0	14.50	3.302
Hallux valgus angle	16.0	49.0	29.34	8.404
Distal metatarsal articular angle	6.2	36.0	15.53	5.287
Metatarsal pronation angle	0.0	26.0	11.71	6.292

Intraobserver reliability for DMAA and MPA measurements was good to excellent, with ICCs of 0.829 for assessor 1 and 0.910 for assessor 2, indicating consistent measurements between assessors. Interobserver reliability was also good (ICC = 0.727).

A weak but statistically significant correlation was found between DMAA and MPA (*r* = 0.337, *P* = .017). There was no significant correlation between DMAA and either the IMA (*r* = 0.046, *P* = .743) or the HVA (*r* = 0.158, *P* = .268).

The mixed effects model revealed that MPA was a significant predictor of DMAA (β = 0.276, 95% CI 0.153-0.399, *P* < .001). Neither HVA nor IMA were significant predictors of DMAA (*P* = .36 and *P* = .53, respectively), suggesting MPA was an independent predictor in this model. There was no significant variance detected between raters (group variance < 0.001), suggesting a high level of agreement and minimal bias between raters.

## Discussion

Our primary aim was to establish whether there was a correlation between DMAA and MPA, and our null hypothesis was that there would be no correlation. We found a statistically significant correlation but weak correlation between these variables suggesting at least some influence of MPA on DMAA. We did not, however, find a correlation between DMAA and other axial plane measurements of hallux valgus. This suggests that DMAA may be at least partly independent of the overall angular severity of deformity and may represent a distinct pathologic feature, rather than a secondary effect of IMA or HVA progression. These results also support the idea that DMAA may be affected by metatarsal pronation.

The DMAA was first described by Piggott in 1960 as a developmental feature of adolescent bunions.^23^ He examined 216 cases and classified first MTP joints into 3 types: congruous, deviated, and subluxated. Congruous joints had well-aligned articular surfaces despite valgus deformity and were considered a variation of normal development. In contrast, deviated and subluxated joints showed progressive structural misalignment and were viewed as pathologic stages. Piggott noted that congruous joints could have DMAA values as high as 28 degrees, whereas subluxation was seen even with angles as low as 19 degrees. He suggested that congruous deformities do not typically progress and may not require treatment in adolescence.

The reliability of the measurement has, however, long been debated, with inconsistent findings reported across studies depending on imaging modality, observer experience, and measurement technique.^3,6-7^ In a 2012 retrospective study, Lee et al^7^ evaluated the reliability of 8 radiographic parameters used in HV assessment. Similar to us, they found the DMAA showed the lowest interobserver reliability (ICC = 0.380, 95% CI 0.149-0.592), compared with higher consistency for hallux valgus and intermetatarsal angles.

The same authors further found that DMAA had strong correlations with both the hallux valgus angle and sesamoid rotation, linking transverse plane deformities with frontal plane pronation.^7^ Other authors have since also questioned the existence of the DMAA as a true deformity, suggesting it may instead be a projection artefact caused by first metatarsal pronation. Chi et al^3^ noted that DMAA appeared to vary with the severity of hallux valgus and between observers. They also observed a small reduction in DMAA following proximal correction of an average of 3.9 degrees. They further emphasised the limitations of DMAA as a consistent and reliable radiographic measurement as it varies with examiners and with severity of hallux valgus angle.

CT provides more consistent and reliable DMAA measurements than plain radiographs. Cruz et al^24^ conducted a prospective study of 77 feet in 43 hallux valgus patients, comparing DMAA measurements on plain radiographs and 3D CT reconstructions. Two observers assessed the DMAA using both methods. Interobserver agreement was significantly higher with 3D CT (concordance correlation coefficient 0.90, *P* < .001) than with radiographs (0.667, *P* < .001). Bland-Altman analysis showed that discrepancies between the 2 methods increased with higher DMAA values and the presence of metatarsal pronation. The study suggests that metatarsal pronation may directly influence radiographic angle measurements, contributing to poor agreement. In our study the interobserver reliability (ICC) was 0.727, showing good reliability between the authors; however, this is less reliable than the other measurements. 3D CT reconstruction is therefore recommended in cases where there is uncertainty about the orientation of the metatarsal head or the accuracy of DMAA measurement. Lalevée et al^19^ looked at 36 hallux valgus and 20 control feet with radiographs and WBCT and found that radiographs overestimated the DMAA by approximately 14 degrees, and that some of the apparent deformity may be due to metatarsal pronation. However, they also found a residual increase of 8.6 degrees in DMAA in the HV group even when accounting for rotation. These findings corroborate our own and suggest that at least some portion of the DMAA may be due to rotation, although some slight structural valgus deformity may still exist.

However, other factors may also play a role in the DMAA, and measurements may also be affected by the inclination of the first metatarsal. Robinson et al^25^ examined this by analysing 34 cadaveric first metatarsals photographed at 5 degrees increments from 30 degrees supination to 30 degrees pronation, with inclinations of 10 degrees, 20 degrees, and 30 degrees. Three masked reviewers measured the DMAA from a total of 39 digital images of each metatarsal. The results demonstrated statistically significant linear increase in apparent DMAA values with greater metatarsal pronation. Inclination also influenced the angle, especially at higher degrees. Despite some interobserver variability, the linear relationship remained stable. This further suggests that DMAA may be an artefact of rotation and inclination of the metatarsal rather than a true anatomical abnormality.

By contrast, metatarsal pronation was first described in the literature in the early 1990s, with initial studies using plain radiographs to assess rotational deformity of the first metatarsal.^26^ Eustace et al^26^ identified morphologic landmarks at the base of the first metatarsal to estimate pronation on standard radiographs, using the movement of the inferior tuberosity in 20 cadaveric first metatarsals positioned at 0 degrees, 10 degrees, 20 degrees, and 30 degrees of pronation. Saltzman et al^27^ further developed this concept by evaluating coronal plane rotation of the first metatarsal using tangential weightbearing radiographs. In recent years, Najefi et al^14-17^ have performed a series of studies examining the MPA in normal feet and patients with HV. Steadman et al^28^ also looked at establishing normative values for metatarsal pronation in asymptomatic feet without hallux valgus deformity. The study found mean first metatarsal rotation values of 2.1 degrees (Saltzman method) and 6.1 degrees (Kim method), with excellent interobserver reliability and intraobserver reliability.^21,26,27^ Najefi et al defined the mean first metatarsal pronation using WBCT as 5.5 ± 5.1 degrees (range, –6 to 25). Interobserver and intraobserver reliability for MPA measurement was excellent (ICC = 0.80 and 0.97, respectively).^17^

Metatarsal pronation is therefore a more established anatomical finding, although the precise origin of the rotational deformity of the first metatarsal is not fully understood. Previous studies have mainly assessed its rotation relative to the ground or second metatarsal, without evaluating the contributions of the navicular or middle cuneiform bones. Geng et al^9^ reported that the first metatarsal-cuneiform joint undergoes dorsiflexion, supination, and internal rotation during loading, with HV patients showing hypermobility across multiple planes. Cychosz et al^29^ found that patients with hallux valgus deformity showed significantly increased first metatarsal pronation compared with controls and had a higher prevalence of flatfoot deformity, which correlated more strongly with metatarsal pronation. Their findings highlight the importance of considering foot type when evaluating pronation in hallux valgus patients. Ota et al^30^ conducted a CT-based 3D analysis of 39 feet and found that torsion of the first metatarsal head relative to its base was significantly greater in HV patients (17.6 ± 7.7 degrees) than in controls (4.7 ± 4.0 degrees), confirming increased axial rotation in HV (*P* < .01). Collectively, these findings emphasise the complex, multiplanar mechanics of HV and the importance of assessing the entire medial column during evaluation and surgical planning.

The literature therefore supports our finding that the DMAA may be at least partly a coronal plane rotational deformity, rather than a pure axial plane deformity. In this setting, however, detection of an abnormal DMAA on plain radiograph may be a cue to considering whether there is a coronal plane deformity and may prompt further cross-sectional assessment.

## Strengths and Limitations

This study presents several strengths and limitations. Among its strengths, the use of WBCT provides accurate 3D imaging, minimising projection errors associated with plain radiographs and increasing the accuracy of DMAA and MPA measurements, as supported by the literature. The study also assessed both intra- and interobserver reliability using ICCs, which were reasonably high (intraobserver ICC = 0.829-0.910; interobserver ICC = 0.727), supporting the reliability of the measurements. It addresses a clinically relevant question regarding the relationship between DMAA and metatarsal pronation, providing valuable guidance for preoperative decision making in hallux valgus correction.

There are some limitations in this study. It is a retrospective study that may have introduced potential selection bias. There is no control group for comparison. The relatively small sample size and being a single-centre study may make it less applicable to wider populations. The study also did not correlate DMAA or MPA with postoperative outcomes, limiting its clinical applicability. Although MPA and DMAA correlated, the correlation was weak, suggesting other factors at play in the DMAA, which this study was not designed to investigate. We have used digitally reconstructed radiographs to measure DMAA. Although this is useful, as it measures what would be seen on a radiograph (and therefore mirrors common practice), it comes with the flaws and drawbacks of radiographic measurements. To establish exactly what proportion of the deformity is rotation vs valgus angulation, a 3D articular plane measurement would be required. However, this requires specialized software and was beyond the scope of this article, but this would be a natural avenue for future research. In addition, the radiographic appearance of where the cartilage sits on the head of the metatarsal can be incorrect, as we are using bony contours rather than actual cartilage mapping. Lastly, while intraobserver reliability was strong, interobserver reliability was slightly lower (although still good), indicating some variability between assessors, particularly with DMAA.

## Conclusion

There was reasonable reliability in measuring DMAA between authors on WBCT although some variations existed between measurements. Despite this, DMAA appeared to modestly increase with increasing metatarsal pronation. The DMAA may therefore be (at least in part) a projection artefact secondary to metatarsal pronation and surgeons should be aware of this during surgical planning, as DMAA values measured on plain radiograph may be misleading. Further work is needed to determine the exact nature of metatarsal pronation and to investigate the effect of correcting pronation during hallux valgus surgery on the DMAA.

## Supplemental Material

sj-pdf-1-fai-10.1177_10711007251412444 – Supplemental material for Distal Metatarsal Articular Angle and First Metatarsal Pronation: A Weightbearing CT Correlation Study Supplemental material, sj-pdf-1-fai-10.1177_10711007251412444 for Distal Metatarsal Articular Angle and First Metatarsal Pronation: A Weightbearing CT Correlation Study by Arvind Vijapur, Mohammed Shaath, Shelain Patel, Nicholas Cullen, Karan Malhotra and Matthew Welck in Foot & Ankle International
